# Bioavailable human metabolites from TOTUM-448 (plant-based formulation) maintain liver cell functionality in a hyperlipidic context that drives MASLD onset

**DOI:** 10.1038/s41598-025-32556-z

**Published:** 2025-12-16

**Authors:** Fabien Wauquier, Vivien Chavanelle, Annie Bouchard-Mercier, Line Boutin-Wittrant, Yolanda F. Otero, Stéphanie Krisa, Josep Valls, Florian Le Joubioux, Bruno Pereira, Véronique Roux, Nicolas Macian, Gisèle Pickering, Véronique Sapone, Murielle Cazaubiel, Auriane Bron, Sébastien Peltier, Stéphanie Blanquet, Pascal Sirvent, Yohann Wittrant

**Affiliations:** 1 Faculté de Médecine, Clinic’n’Cell SAS, 28 place Henri Dunant, Clermont-Ferrand, 63001 France; 2 Valbiotis, 20 rue Henri et Gilberte Goudier, Riom, 63200 France; 3 Université de Bordeaux-INRAE-INP-ISVV, 210 Chem. de Leysotte, Villenave-d’Ornon, 33140 France; 4 MetaboHUB, Bordeaux Metabolome, 210 Chem. de Leysotte, Villenave-d’Ornon, 33140 France; 5 Valbiotis, Zone Industrielle des 4 Chevaliers, Bâtiment 12 F, Rue Paul Vatine, Perigny, 17180 France; 6 CIC INSERM 1405/Plateforme d’Investigation Clinique CHU Gabriel Montpied, Clermont-Ferrand, 63000 France; 7 Biostat Unit, DRCI, CHU, Clermont-Ferrand, 63000 France; 8 Digestive Environment and Health, Université Clermont Auvergne, UMR 454 MEDIS UCA-INRAE, Microbiology, Clermont-Ferrand, France; 9 International associated laboratory Host Microbes Interactions in the Human Gut (HOMIGUT), Clermont-Ferrand, France; 10 Center for Microbial Ecology and Technology (CMET), Department of Biotechnology, Ghent University, Ghent, Belgium; 11 INRAE, UMR 1019, UNH, Clermont-Ferrand, 63000 France

**Keywords:** Clinical trial, Ex vivo, MASLD, Plant extract, Human metabolites, Human hepatocytes, Oxidative stress, Lipotoxic stress, Biochemistry, Cell biology

## Abstract

Lipotoxic and inflammatory environment drives metabolic dysfunction-associated steatotic liver disease (MASLD) onset. As most conventional treatments present adverse side effects, alternative options such as preventive nutritional interventions have been developed, though further clinical validation is needed. In this study, we conducted an innovative ex vivo clinical investigation to examine how circulating metabolites generated after oral intake of TOTUM-448 (a plant-based, polyphenol-rich formulation) may influence hepatocyte function. UHPLC-MS/MS analysis confirmed and characterized the bioavailable polyphenol metabolites present in human serum. This metabolite-enriched serum was further used to treat HepG2 hepatocytes, with or without palmitate pretreatment (250 µM). The effects of TOTUM-448–derived metabolites on hepatocytes were evaluated by monitoring cell viability, lipid metabolism, inflammation, oxidative stress, and endoplasmic reticulum (ER) stress, all of which are central features of MASLD. Treated hepatocytes exhibited resistance to palmitate-induced lipotoxic stress, showing reduced intracellular lipid accumulation. TOTUM-448–derived metabolites also prevented the palmitate-induced upregulation of inflammatory gene expression. Additionally, while palmitate strongly upregulated CHOP and XBP1 mRNA expression as well as ATF6 and Caspase-3 activities, the presence of TOTUM-448–derived metabolites restored these ER stress markers to normal levels.

**Supplementary Information:**

The online version contains supplementary material available at 10.1038/s41598-025-32556-z.

## Introduction

Chronic liver disease is a social and economic burden^1^. Among the most common liver disorders is metabolic dysfunction–associated steatotic liver disease (MASLD), redefined in 2023 and previously described as non-alcoholic fatty liver disease (NAFLD)^2^. Its prevalence is estimated at about 30% of the adult population worldwide^3–5^. More precisely, the prevalence can reach up to 90% in individuals with obesity and 60% in those with diabetes^1^^,^^6^. However, MASLD is not limited to overweight individuals, as approximately 15% of patients present with a normal body weight^7^.

MASLD is characterized by the association of excessive fat accumulation in liver with one or more metabolic risk factor such as overweight/obesity, type 2 diabetes, metabolic syndrome, hypertriglyceridemia and hypercholesterolemia^2^. This steatosis results from the accumulation of lipids in liver parenchymal cells and triggers hepatic inflammation and fibrosis. Over time, it leads to cirrhosis and its associated complications^8^. Consequently, MASLD is associated with elevated risks of cancer and cardiovascular mortality^9,10^ and ranks as the second most frequent indication for liver transplantation in the United States and Europe^11,12^.

In this context, strategies promoting hepatic lipid clearance are of particular interest. Different pharmacological approaches have been studied, including statins, SGLT-2 inhibitors, GLP-1 agonists, pioglitazone, vitamin E, Resmetirom and Semaglutide (Wegovy)^3,13^^,^^14^^,^^15^. The latter two, Resmetirom, a liver-directed thyroid hormone receptor beta-selective agonist, and Wegovy, a glucagon-like peptide-1 receptor agonist, have recently been approved by FDA for MASH treatment (March 2024 and August 2025, respectively)^16–18^. However, these treatments designed to cure rather than to prevent, evidence side effects (e.g., muscle related issues for statins; gastrointestinal adverse events for both resmetirom and semaglutide)^14,15^ and may be quite expensive for patients and healthcare systems^19^. These observations highlight the necessity for safer and preventive therapeutic alternatives. Lifestyle modifications, including dietary changes and regular physical activity constitute the first-line treatment, while nutritional and nutraceutical strategies have emerged as a major area of therapeutic innovation^1^.

Among natural bioactives of interest, polyphenols are plant compounds known for their antioxidant and anti-inflammatory properties. Notably, over half of the research on their role in MASLD has been published in the past five years, underscoring the increasing focus on nutritional strategies in this area. Yet, only about 7% of these investigations have been conducted at the clinical level. Certain plant extracts are already known for their hepato-protective properties. For example, artichoke leaf extract (*Cynara scolymus*) is widely described in the literature for its choleretic, hepatoprotective^20^ and lipid metabolism regulation properties^21–24^. However, these approaches are limited by a lack of robust clinical evidence, and optimizing their benefits, particularly through potential nutritional synergies, requires further clinical validation.

TOTUM-448 is a patented polyphenol-rich blend of 5 different plant extracts (olive leaf (*Olea europaea*), bilberry (*Vaccinium myrtillus*), artichoke leaf (*Cynara scolymus*), chrysanthellum (*Chrysanthellum indicum subsp. afroamericanum* B.L. Turner), black pepper (*Piper nigrum*)) and choline, with potential synergistic properties on liver function. The aim of this work was to investigate whether and how this combination can maintain, preserve, or even improve hepatocyte metabolism in a lipotoxic context.

To address this working hypothesis, we collected human serum containing circulating metabolites resulting from TOTUM-448 ingestion and, following Clinic’n’Cell’s patented methodology^24–31^, we evaluated ex vivo their effect on the function and responses of human hepatocytes under a lipotoxic condition.

## Results

### Kinetic analysis of circulating metabolites detected in human serum following from TOTUM-448 ingestion

**Fig. 1:**
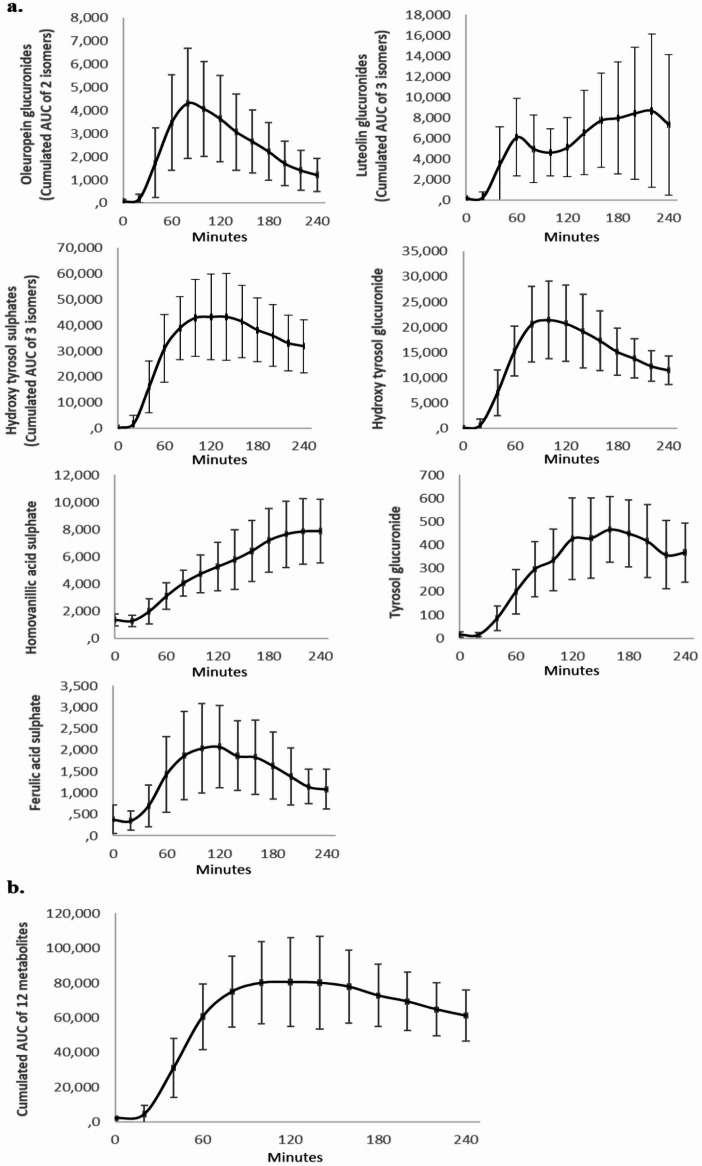
Metabolomic profiles in human serum following TOTUM-448 ingestion. **A**: Circulating metabolites resulting from TOTUM-448 ingestion were determined using Ultra-High Performance Liquid Chromatography-Mass Spectrometry (UHPLC-MS/MS).Twelve detectable circulating human metabolites are presented (2 oleuropein glucuronide isomers, 3 luteolin glucuronide isomers, 1 ferulic acid sulfate, 3 hydroxytyrosol sulfate isomers, 1 hydroxytyrosol glucuronide, 1 tyrosol glucuronide, and 1 homovanilic acid sulfate). **B**: Cumulative absorption for each metabolite family. AUC: Area under curve. *n* = 10 human volunteers.

TOTUM-448 was ingested and digested by fasted healthy volunteers, and the presence of circulating metabolites in the bloodstream was monitored over time using a kinetic approach to determine their absorption profile. We found 12 detectable circulating human metabolites, including 2 oleuropein glucuronide isomers, 3 luteolin glucuronide isomers, 1 ferulic acid sulfate, 3 hydroxytyrosol sulfate isomers, 1 hydroxytyrosol glucuronide, 1 tyrosol glucuronide, and 1 homovanilic acid sulfate (Fig. 1A). The Tmax ranged from 40 min to 160 min post-absorption depending on the targeted metabolite and the volunteer. To get a representative viewpoint of the diversity of the metabolites detected, we plotted, for each time point, the cumulative absorption for each metabolite family. The resulting curve rapidly reached a peak between 80 and 140 min (Fig. 1B). Thus, to ensure the collection of serum fractions enriched with the highest diversity of metabolites, the 100 min time point (post-ingestion) was chosen for blood sampling during the second clinical phase.

## Viability of HepG2 hepatocytes exposed to naïve or metabolite-enriched human serum

**Fig. 2:**
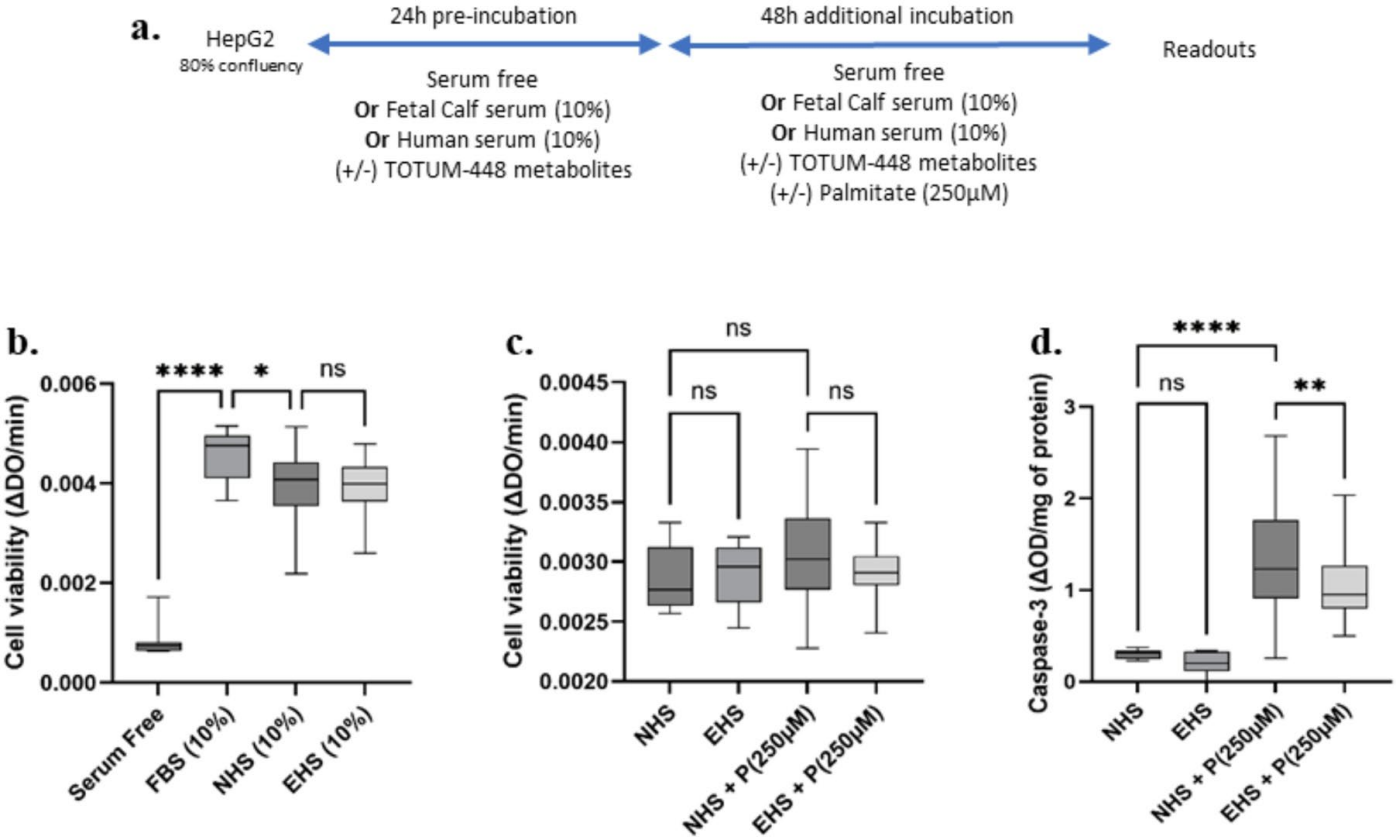
Cell culture experimental design and validation of the ex vivo procedures (Human hepatocytes, HepG2). (**A**) Protocol design. (**B**) Validation of the use of human serum for human hepatocyte cultures. Cell viability was determined using a XTT-based method. Fetal Bovine Serum (FBS) was used as the reference. (**C**) Impact of palmitate on hepatocyte viability. (**D**) Caspase-3 activity. NHS: Naïve human serum; EHS: Enriched human serum (serum collected at Cmax 100 min and containing circulating metabolites resulting from TOTUM-448 ingestion); P(250µM): palmitate 250µM. The control vehicle was set according to the palmitate preparation, a 10% w/w BSA solution was added to an equivalent volume of ethanol to match the final palmitate solution. Measurements were realized in hexaplicate (XTT) or triplicate (caspase-3 activity) for each volunteer (*n* = 10 human volunteers). Boxes indicate median and interquartile range (lower and upper), while whiskers indicate minimum and maximum *: *p* < 0.05; **: *p* < 0.01; ****: *p* < 0.0001; ns: *p* > 0.05.

The final objective of this clinical ex vivo approach was to investigate whether and how these bioavailable human metabolites may preserve human hepatocyte function (in comparison with naïve serum) in a lipotoxic context mimicking MASLD. Therefore, blood was sampled out from the subjects before the ingestion of TOTUM-448 (naïve fraction), and at 100 min post ingestion (enriched fraction) for ex vivo investigations. Cell culture procedures were set as presented in Fig. 2A. Hepatocytes were pre-incubated in the presence of either naïve serum (NHS) or TOTUM-448 enriched serum (EHS) for 24 h before exposure to 250 µM palmitate for 48 h, resulting in a total serum incubation of 72 h.

To ensure the biological soundness of this ex vivo approach, we first checked that human serum (either naïve or enriched) had no adverse impact on cell growth and viability (Fig. 2B). As expected, the absence of fetal calf serum stopped the cell proliferation, while its presence allowed normal cell behavior. The presence of human serum slightly limited cell growth but there was no significant difference between the naïve or the enriched fraction (Fig. 2B). The presence of palmitate had no impact on this parameter (Fig. 2C). However, when looking more precisely into the pro-apoptotic mechanisms, we found that palmitate markedly induced caspase-3 activity (Fig. 2D). This induction was significantly limited in the presence of the enriched serum fraction (group EHS-P compared to NHS-P; −31%, *p* ≤ 0.01, Fig. 2D).

## TOTUM-448 human metabolites limit fat accumulation in human hepatocytes

**Fig. 3:**
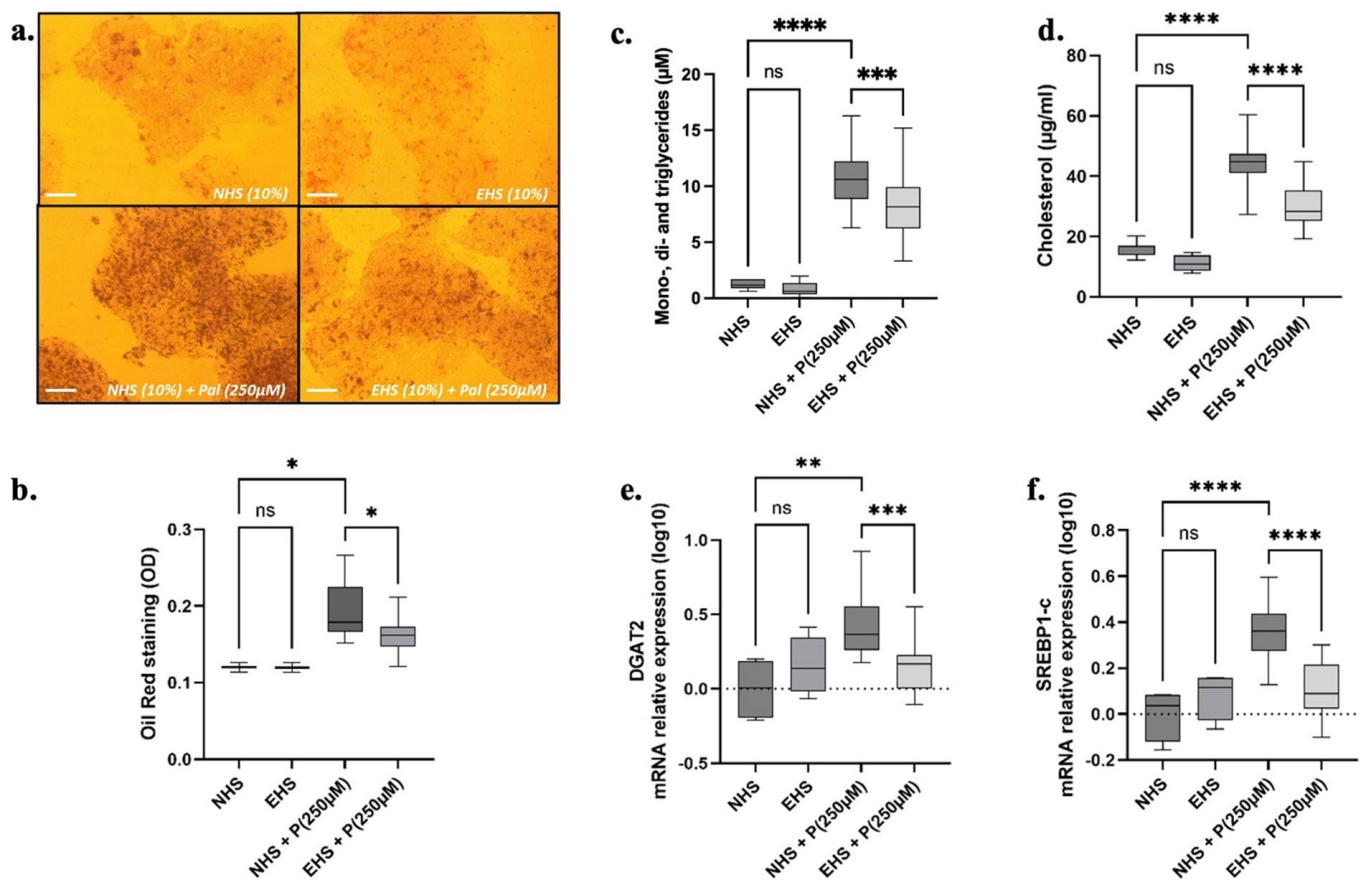
Influence of TOTUM-448 metabolites on lipid storage in human hepatocytes. (**A**) Red oil staining. (**B**) Red oil staining quantification. (**C**) Intracellular cumulated mono-, di- and triglyceride content. (**D**) Intracellular cholesterol content. E and F: mRNA relative expression of DGAT2 and SREBP1c (RTqPCR). NHS: Naïve human serum; EHS: Enriched human serum (containing circulating metabolites resulting from TOTUM-448 ingestion); P(250µM): palmitate 250µM. The control vehicle was set according to the palmitate preparation, a 10% w/w BSA solution was added to an equivalent volume of ethanol to match the final palmitate solution. Measurements were realized in triplicate for each volunteer (*n* = 10 human volunteers). Boxes indicate median and interquartile range (lower and upper), while whiskers indicate minimum and maximum *: *p* < 0.05; **: *p* < 0.01; ***: *p* < 0.001; ****: *p* < 0.0001; ns: *p* > 0.05.

Hepatocytes were subjected to 250 µM palmitate to reproduce MASLD-associated lipotoxicity, in presence of either naïve or enriched serum, to determine if metabolites could maintain cellular function under these conditions. Red oil staining revealed that the presence of palmitate dramatically promoted lipid storage in hepatocytes while neither NHS nor EHS impacted lipid accumulation. Pre-incubation of hepatocytes with EHS before palmitate exposure resulted in a significant reduction of Red-Oil staining (Fig. 3A and B). To gain deeper insight, we analyzed the lipid species involved in this accumulation. Consistent with the results obtained with Red-Oil staining, palmitate exposure significantly stimulated the accumulation of esterified fatty acids (under the form of mono-, di and triglycerides) and cholesterol in human hepatocytes. The presence of TOTUM-448 metabolites in group EHS-P significantly prevented lipid storage, compared to NHS-P (mono-, di-, triglycerides − 27%%, *p* ≤ 0.001; cholesterol − 52%, *p* ≤ 0.0001, Fig. 3C and D). Finally, we investigated hepatocytes transcriptomic activity of genes related to lipid metabolism. Exposure to palmitate led to a significant increase in DGAT2 and SREBP1c expression (Fig. 3E and F). Co-incubation with EHS fraction (group EHS-P) significantly blunted this effect, reducing DGAT2 and SREBP1c levels by 64% (*p* ≤ 0.001) and 68% (*p* ≤ 0.0001), compared to NHS-P, respectively.

### Limited contribution of oxidative and inflammatory stress modulation to the effects of TOTUM-448 metabolites in human hepatocytes

**Fig. 4:**
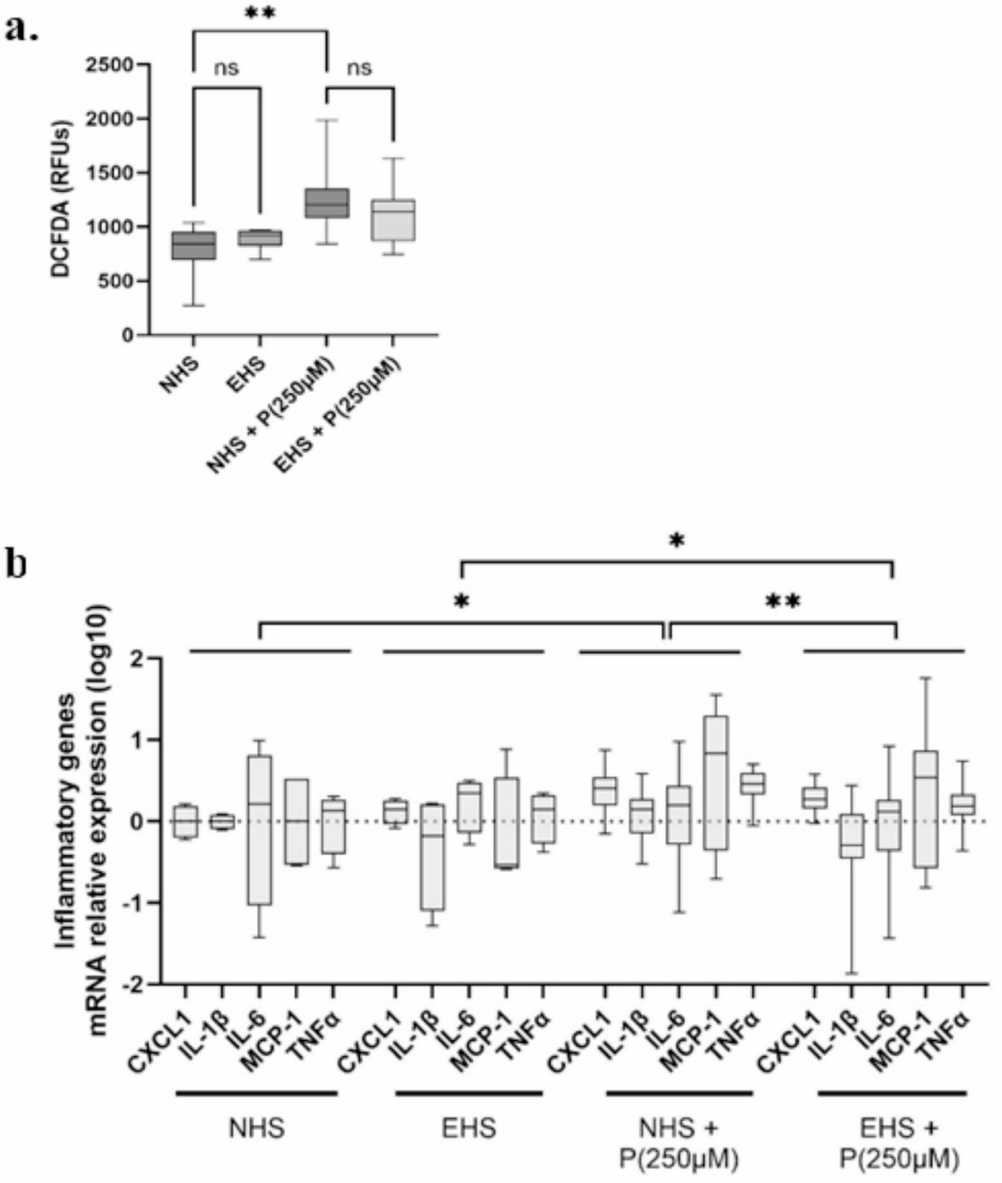
Influence of TOTUM-448 human metabolites on redox and inflammatory status in human hepatocytes HepG2. **A**: DCFDA staining (quantification expressed as the mean green pixel value); **B**: mRNA relative expression of CXCL1 IL-1β, IL-6, MCP-1 and TNFα. NHS: Naïve human serum; EHS: Enriched human serum (containing circulating metabolites resulting from TOTUM-448 ingestion); P(250µM): palmitate 250µM. The control vehicle was set according to the palmitate preparation, a 10% w/w BSA solution was added to an equivalent volume of ethanol to match the final palmitate solution. Measurements were realized in triplicate for each volunteer (*n* = 10 human volunteers). Boxes indicate median and interquartile range (lower and upper), while whiskers indicate minimum and maximum *: *p* < 0.05; **: *p* < 0.01; ns: *p* > 0.05.

Along with lipotoxicity, the development of inflammation and oxidative stress is a potent driver of altered hepatocyte function and subsequent MASLD onset. Using a DCFDA probe, we showed that palmitate exposure promoted ROS production (Fig. 4A). The presence of TOTUM-448 metabolites in group EHS-P tended to limit this production (*p* > 0.05). Regarding the inflammatory status, while only statistical trends appeared when expressions of the targeted genes were considered separately (suppl. figure S1), 2-way ANOVA revealed a significant group effect (*p* = 0.0008) and significant differences in *post hoc* comparisons of overall inflammatory gene expression between groups NHS and NHS + P (250µM), *p* < 0.05, EHS and EHS + P(250µM), *p* < 0.05, and NHS + P (250µM) and EHS + P (250µM), *p* < 0.01, Fig. 4B. This suggests that palmitate consistently induced inflammatory gene expression while the presence of Totum-448 metabolites significantly limited this induction. Nonetheless, Totum-448 metabolites did not fully prevent the induction, as the inflammation markers in EHS-P group remained significantly different from the EHS control group.

## TOTUM-448 human metabolites prevent lipotoxicity-induced ER stress in human hepatocytes

**Fig. 5:**
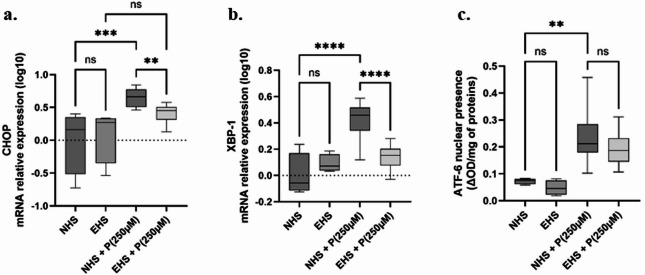
Influence of TOTUM-448 human metabolites on ER stress level and UPR markers in a lipotoxic environment. **A** and **B**. mRNA relative expression of CHOP and XBP-1, respectively. **C**. ATF-6 nuclear presence. NHS: Naïve human serum; EHS: Enriched human serum (containing circulating metabolites resulting from TOTUM-448 ingestion); P(250µM): palmitate 250µM. The control vehicle was set according to the palmitate preparation, a 10% w/w BSA solution was added to an equivalent volume of ethanol to match the final palmitate solution. Measurements were realized in triplicate for each volunteer (*n* = 10 human volunteers). Boxes indicate median and interquartile range (lower and upper), while whiskers indicate minimum and maximum **: *p* < 0.01; ***: *p* < 0.001; ****: *p* < 0.0001; ns: *p* > 0.05.

Abnormal lipid accumulation in steatotic liver coincides with perturbed endoplasmic reticulum (ER) proteostasis in hepatocytes. Therefore, we checked for Unfolded Protein Response (UPR) markers in palmitate stressed hepatocytes. As expected, palmitate markedly and significantly promoted both CHOP and XBP-1 gene expression (Fig. 5A and B). Such induction was abolished when cells were treated with TOTUM-448 metabolites in EHS-P, compared to NHS-P (−36%, *p* ≤ 0.01 and − 66%, *p* ≤ 0.0001 for CHOP and XBP1, respectively). Neither the naïve (NHS) nor the enriched human serum (EHS) fraction had any impact on those markers in the absence of a lipotoxic context. To support these data, we analyzed ATF-6 activity as the result of the presence of ATF-6 protein in the nucleus. ATF-6 activity was significantly increased following palmitate stress in NHS-P. In contrast, TOTUM-448 metabolites (EHS-P) tended to inhibit this phenomenon, although this result did not reach statistical significance (−29%, *p* = 0.2, Fig. 5C).

## Discussion

In this manuscript, we first demonstrated that the ingestion of TOTUM-448 (1) led to bioavailable circulating metabolites. When incubated with human hepatocytes, these metabolites attenuated palmitate-induced intracellular storage of several lipids including (2) esterified fatty acids (under the form of mono-, di and triglycerides) (−27%, *p* ≤ 0.001) and (3) cholesterol (−52%, *p* = 0.0001), while inhibiting the gene expression of (4) DGAT2 (−64%, *p* ≤ 0.001) and (5) SREBP1-c (−68%, *p* ≤ 0.0001). In addition, TOTUM-448 metabolites also blunted (6) palmitate-induced inflammatory gene expression (*p* ≤ 0.01). The analysis of ER stress markers showed that, while palmitate exposure potently induced (7) CHOP, (*p* ≤ 0.001) and (8) XBP1 mRNA expression (*p* ≤ 0.0001), (9) ATF-6 activity (*p* = 0.0053) and (10) Caspase-3 activity (*p* ≤ 0.0001), the presence of TOTUM-448 metabolites normalized these markers (−36%, *p* ≤ 0.01; −66%, *p* ≤ 0.0001 and − 31%, *p* ≤ 0.01, for CHOP, XBP1 and Caspase-3, respectively; a trend was observed for ATF-6 (−29%, *p* = 0.2)).

Each volunteer was exposed once per clinical phase to 4284 mg of TOTUM-448 representing an approximative global amount of 372 mg of polyphenols (see suppl. Table S1 for chemical characterization of TOTUM-448), making it relevant when considering the recommended daily dose for polyphenol intakes (1 g). Additionally, the dose in this work matches that of our parallel clinical trial (currently underway, NCT06704321), where it is being evaluated for long-term human supplementation.

Following Totum-448 ingestion, we were able to detect 12 different circulating metabolites including 2 oleuropein glucuronide isomers (olive leaf^32^, 3 luteolin glucuronide isomers (metabolites that may derive from luteolin-7-O-glucoside, luteolin-7-O-glucuronide and luteolin presents in *chrysanthellum*, in both artichoke and olive leaves^33^, 1 ferulic acid sulfate (metabolite that may derive from either chlorogenic acid present in artichoke leaf and, *chrysanthellum* or caffeoylquinic acid also present in artichoke leaf^34,35^, 3 hydroxytyrosol sulfate isomers, 1 hydroxytyrosol glucuronide, 1 tyrosol glucuronide, 1 homovanilic acid sulfate (metabolites that may derive from both hydroxytyrosol and oleuropein present in olive leaf^32^. These metabolomic analyses evidenced a clear and diverse bioavailability of the components of the blend and supported the rationale for further investigation of the bioactivity of the serum.

We recruited male healthy volunteers with no treatment or pathology, ageing from 18 to 35, with a BMI from 20 to 28, and with normal kidney and liver functions. In our clinical ex vivo approach, volunteers should be seen as metabolites producers rather than patients expecting health benefits with measurable clinical scores. Thus, this protocol was designed to collect circulating metabolites in a standardized way. The collected serum, either naïve (control baseline) or enriched (with circulating metabolites of interest) are further used/incubated on cell cultures according to the patent DIRV#18–0058 (written invention disclosure by the French National Institute for Agronomic, Food and Environment Research INRAE) to investigate a wide range of cell activities/markers that represent the final readout/score. In this study we targeted hepatocytes as a major protagonist in lipid metabolism and MASLD onset.

Hepatocytes are responsible for glucose, xenobiotic, and lipid metabolism. In this culture model, palmitate consistently promoted lipid accumulation in hepatocytes including esterified fatty acids (under the form of mono-, di and triglycerides) and cholesterol mimicking the onset of MASLD. The presence of metabolites resulting from TOTUM-448 ingestion potently prevented this phenomenon. From a mechanistic point of view, these results align with the downregulation of the gene expression of DGAT2, an enzyme involved in triglycerides synthesis^36^ and SREBP1c, involved in the *de novo* lipogenesis in the liver^37^. Besides, these results are fully consistent with published observations regarding the effect of the different components of TOTUM-448 formulation studied separately. Indeed, in patients with diagnosed MASLD, supplementation with artichoke leaf extracts (*Cynara scolymus L*., present in Totum-448) (600 mg daily for 9 weeks) reduced liver size and decreased serum total cholesterol and triglyceride concentrations^38^. In a high-fat diet rat model, artichoke leaf extract supplementation limited hepatic steatosis^39^. Additionally, a clinical study on a cohort of steatotic patients showed that supplementation with *Chrysanthellum americanum* extract (another ingredient of Totum-448) decreased fibrosis and hepatic steatosis^40^.

In MASLD, the lipotoxic context fuels inflammation and oxidative stress by stimulating ROS production, pro-inflammatory cytokine release and by promoting the recruitment of immune cells. Interestingly, our cellular model replicates these events, as we showed that palmitate exposure increased DCF-DA staining and inflammatory gene expressions. Of note, these detrimental effects were blunted in hepatocytes incubated with TOTUM-448-enriched serum. This observation must be tempered by the fact that the induction of the expression of CXCL1, IL-1β, IL-6, MCP-1 and TNFα did not reach significance when analyzed separately, suggesting a global effect on inflammatory status rather than a specific effect on a target gene. Consistently, in a recent randomized, controlled clinical study, Guo et al. demonstrated that NLRP3 inflammasome activation is significantly increased in patients with MASLD, but that this activation can be strongly repressed by anthocyanins (provided here by blueberry extract, *Vaccinium myrtillus L*^41^.). Additionally, several polyphenols present in the enriched serum have already demonstrated antioxidant properties through scavenging of free radicals or inhibition of oxidative enzymes^42^^,^^43^including oleuropein in a similar HepG2 model^44^. Preclinically, olive leaf extracts have shown anti-inflammatory, antioxidant, and antifibrotic effects^45^. Finally, a 2021 randomized double-blind clinical study shows that supplementation with olive leaf extract (*Olea europaea L*.), characterized by its oleuropein and hydroxytyrosol content, shows a reduction in oxidized LDL^46^.

Hepatocytes are involved in *de novo* lipid synthesis as well as lipid metabolism, making the smooth and rough endoplasmic reticulum (ER) crucial for these functions. Dysregulated lipid metabolism, inflammation, or oxidative stress can thus be both a cause and a consequence of ER dysfunction. As a consequence, a vicious cycle is established that triggers the UPR (Unfolded Protein Response) and activates IRE1, PKR-like ER kinase (PERK), and ATF6 to restore ER homeostasis^47^. Notably, PERK activation induces an antioxidant response^48,49^, while both PERK and ATF6 contribute to liver recovery, as demonstrated by knockout studies^50–52^.

In this study, the influence of the TOTUM-448 metabolites on ER-stress management seems to be the cornerstone. In our hepatocytes, palmitate exposure enhanced both XBP-1^53^^,^^54^ and CHOP^55^ expressions and significantly stimulated ATF-6 activity. XBP-1 and CHOP upregulation result from the activation of IRE1 and PKR-like ER kinase (PERK), respectively. This upregulation is fully consistent with the literature data. The association between chronic ER and steatotic liver was previously established in both obese mouse models (genetic and high-fat diet models) and obese patients^56^. In parallel, palmitate exposure also significantly enhanced caspase-3 activity in HepG2 cells. Surprisingly, palmitate did not affect hepatocyte viability after 72 h, a parameter typically considered a hallmark of lipotoxicity. This apparent lack of effect may reflect a lipotoxic stress level sufficient to elicit measurable cellular responses without compromising viability within the timeframe of the experiment. Yet, the absence of cell death during the study period reduces potential bias in data interpretation. The increase in caspase-3 activity, however, was consistently described in liver biopsies from patients suffering from MASH^57^. In line with this, in rats fed a high fat diet, CHOP expression and the active form of caspase-3 were found increased in liver^58^. Interestingly in our work, the presence of metabolites from TOTUM-448 potently limited this palmitate-induced ER stress and normalized the gene expression of these markers (−36%, *p* ≤ 0.01; −66%, *p* ≤ 0.0001 and − 31%, *p* ≤ 0.01, for CHOP, XBP1 and Caspase-3, respectively; while a trend was observed for ATF-6 (−29%, *p* = 0.2)) supporting the relevance of this nutritional strategy to manage MASLD. The protective effects of the serum enriched with TOTUM-448-derived metabolites against palmitate-induced endoplasmic reticulum (ER) stress may, at least in part, be attributed to the action of specific polyphenols known to be present in the plant extracts composing TOTUM-448. Several of these compounds have already been reported to mitigate ER stress. For example, hydroxytyrosol, a major compound of olive leaf extract, has been shown to protect HepG2 cells from tunicamycin-induced ER stress^59^. Similarly, luteolin, found in artichoke and *Chrysanthellum* extracts, was reported to prevent palmitate-induced ER stress, autophagy and apoptosis in AML12 hepatocytes^60^. In addition, chlorogenic acid, which is present in artichoke leaf extract, demonstrated protective effects against palmitate-induced ER stress in primary rat hepatocytes^61^.

**Fig. 6:**
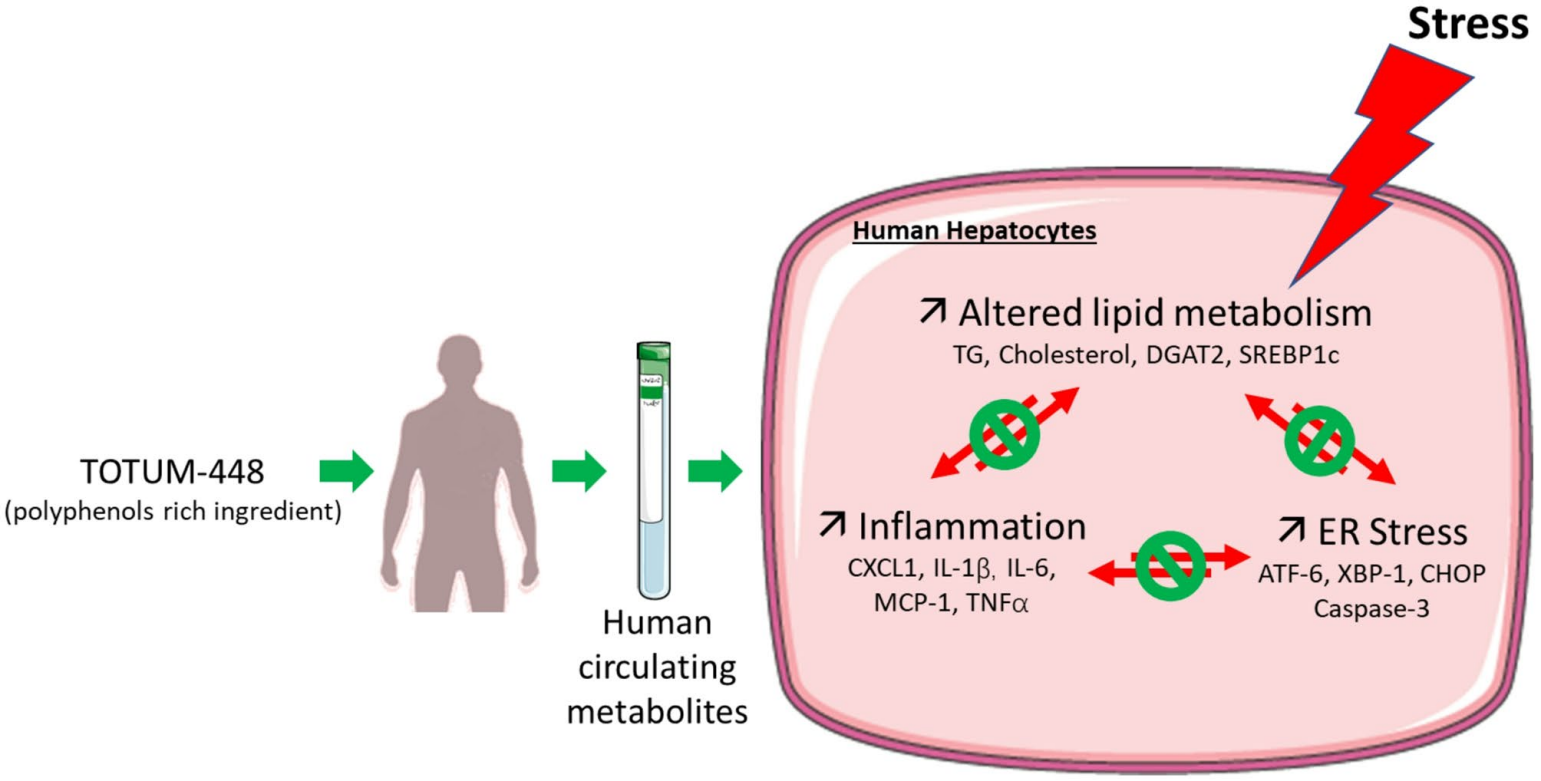
Bioavailable Human Metabolites from TOTUM-448 (Plant-Based, Polyphenol-Rich Ingredient) Maintain Liver Cell Functionality in a Lipotoxic Context that Drives MASLD Onset.

Inflammation, oxidative stress and ER stress being widely imbricated^62,63^ we are facing a chicken-and-egg situation and the mode of action by which TOTUM-448 metabolites confer hepatoprotection remains unclear, as they may either improve hepatocyte function and lipid metabolism by preserving ER homeostasis, or ER stress may decrease as a result of enhanced lipid metabolism. Indeed, it has been reported that caloric restriction improves steatosis and alleviates ER stress^64^ while normalization of ER stress markers was correlated with reduced hepatic lipid content^65^. ER is the major site of lipid synthesis in hepatocytes including triglyceride synthesis and storage. Indeed, SREBP1c and SREBP2, transcription factors responsible for fatty acid and sterol synthesis respectively, together with the enzyme diacylglycerol acyltransferase (DGAT), are localized to the ER and play key roles in regulating and catalyzing de novo lipogenesis^47^. Accordingly, TOTUM-448 metabolites were found to reduce SREBP1c and DGAT2 expression to blunt the vicious cycle of MASLD and promote hepatocyte homeostasis. Notably, the metabolites had no effect on their own but showed protective effects in the presence of palmitate, supporting the preventive role of this ingredient in maintaining hepatocyte function and homeostasis (Fig. 6).

We acknowledge several limitations to this study. First, the type of fatty acid used to induce lipotoxicity may play a role. In recent lipidomic studies using in vitro models of lipotoxicity in hepatocytes (HepaRG and HuH7), palmitic acid (PA) was found to induce a more severe phenotype than oleic acid with greater inflammation and oxidative stress, accompanied by specific changes in the lipidome^66,67^. In this work, we chose to use only palmitic acid. Since saturated and unsaturated fatty acids modulate lipid metabolism differently in these models (a statement that is particularly true for triglycerides metabolism), further investigations with a combination of different type of fatty acids would increase the physiological relevance of the observed hepatoprotective properties of TOTUM-448. On a different note, although HepG2 cells provide a well-characterized and reproducible hepatocyte model for assessing lipotoxicity-associated mechanisms, they remain a cell line derived from a hepatocellular carcinoma and hence differ from primary hepatocytes in many physiological features including metabolic profile, inflammatory cascade and lipotoxic sensitivity. HepG2 cells display limited activation of inflammatory pathways compared with primary hepatocytes or co-culture systems including immune cells. Additional investigations in primary hepatocytes would be required to reach a more comprehensive view. However, our study primarily aimed to investigate hepatocellular responses to lipotoxic stress rather than to model the full inflammatory cascade. Another limitation of this study lays in the fact that oxidative stress was assessed using DCFDA staining, which reflects total cellular ROS levels. Although this approach does not specifically distinguish mitochondrial ROS, it provides an integrated measure of redox imbalance under palmitic acid exposure.

In conclusion, this ex vivo clinical trial demonstrates for the first time that TOTUM-448–derived metabolites protect hepatocytes from lipotoxic stress by modulating lipid metabolism, inflammation, and ER stress, highlighting the potential of TOTUM-448 formulation as a preventive strategy for managing early stages of MASLD.

### Methods

#### Study product

VALBIOTIS has developed TOTUM-448 (priority French patent: FR1460064), a food ingredient composed of the combination of different plant extracts aforementioned, and choline. The selection of the extracts was originally driven by published data regarding their possible support in preventing liver conditions. The formulation of the product meets the requirements of European regulations relating to food supplements (European directive n°2002/46/EC and French decree n°2006 − 352 of March 20, 2006 which transposes it).

## Ethics preclinical study

The study was conducted in accordance with the Declaration of Helsinki of 1975 (https://www.wma.net/what-we-do/medical-ethics/declaration-of-helsinki (accessed on November, 12th 2021)) revised in 2013. The human study was approved by the French Ethical Committee (NCT06047847/N° ID RCB: 2023-A00579-36/Comité de Protection des Personnes CPP Est III; approved 25th July 2023). The volunteers were informed of the objectives and the potential risks of the present study and provided their written informed consent before they participated in the study.

### Human study design and Pharmacokinetic of absorption

Ten healthy men (age: 24.5 years old, +/− 3.48; BMI: 23.35 kg/m2, +/−1.72; >60 kg) were enrolled for this study. Only male healthy volunteers with no treatment or pathology, aged from 18 to 35, BMI ranged from 20 to 28, with normal kidney and liver functions were recruited. They were checked for normal blood formulation, renal (urea and creatinine), and liver functions (aspartate aminotransferase (AST), alanine aminotransferase (ALT), and gamma-glutamyltransferase (GGT) activities). Serum samples from all participants were collected in plain tubes. Unfortunately, female volunteers were not to be recruited to avoid hormonal variation and subsequent bias.

The first phase of this clinical project was dedicated to determine TOTUM-448 metabolites absorption profile. Prior to receiving the tested product, volunteers were fasted for 12 h before being given 4284 mg of TOTUM-448 in the form of eight capsules. Nine milliliters of venous blood (median cubital vein) were collected before the ingestion of TOTUM-448 and then every 20 min for 240 min after the ingestion of the ingredient. Collected serum was aliquoted in sterile tubes and then stored until analyses at − 80 °C. Location of storage: Clinical Investigation Center – Inserm 1405, University Hospital of Clermont-Ferrand. This dedicated research department is fully compliant with regulatory and ethical clinical obligations (certification according to the French standard NF S 96900).

Circulating metabolites resulting from TOTUM-448 ingestion were quantified and characterized by ultra-high performance liquid chromatography with tandem mass spectrometry (UHPLC-MS/MS). Upon characterization of the absorption profile, volunteers underwent a second visit to the clinical center for the second phase of the project in order to collect serum containing TOTUM-448 metabolites at Tmax/Cmax. Context was similar to phase 1, namely, prior to receiving the tested product, volunteers were fasted for 12 h before being were given 4284 mg of TOTUM-448 (eight capsules). Forty-eight milliliters of venous blood were collected before the product ingestion as a control baseline naïve serum. Then, at Cmax in the post-absorptive phase, forty-eight milliliters of enriched blood was collected. Collection, aliquoting and storage conditions were similar to phase 1.

### Phenolic compounds extraction from serum

To extract phenolic compounds from serum, 0.9 mL of serum was combined with 2.7 mL of 100% methanol and mixed for one minute. The mixture was then centrifuged at 20,000× g for 15 min at room temperature. The supernatant was collected in separate tubes and evaporated to dryness using a SpeedVac Concentrator (Thermo Fisher Scientific, Illkirch, France). The dried material was reconstituted in 80 µL of a 50:50 methanol/water solution. After agitation for one minute, ultrasonication for one minute, and centrifugation at 20,000× g for 15 min at room temperature, the supernatant was stored at − 20 °C until analysis by ultra-high performance liquid chromatography with tandem mass spectrometry detection.

### Ultra-High performance liquid Chromatography-Mass spectrometry (UHPLC-MS/MS)

Phenolic compounds of serum were analyzed using a 1260 Infinity UHPLC system (Agilent Technologies) coupled to a 6430 triple quadrupole mass spectrometer (Agilent Technologies, Les Ulis, France). Four microliters of the sample were injected into a Zorbax SB-C18 column (2.1 × 100 mm, 1.8 μm) (Agilent Technologies, Les Ulis, France). The mobile phase consisted of two solvents: solvent A (water/formic acid 99.9:0.1, v/v) and solvent B (acetonitrile/formic acid 99.9:0.1, v/v), with a flow rate of 0.3 mL/min. The gradient for solvent A was as follows: 0 min 1% B, 2 min 5% B, 3 min 25% B, 6 min 25% B, 8 min 40% B, 11.5 min 95% B, 14 min 95% B, and 16 min 1% B. The MS/MS parameters were set to negative ion mode, with a capillary tension of 3000 V, a nebulizer pressure of 15 psi, a dry gas flow rate of 11 L/min, a dry temperature of 350 °C, and acquisition in multiple reaction monitoring (MRM) mode (Supplementary Table S2). Data were processed using MassHunter software (Agilent Technologies).

### Human hepatocyte cultures

The HepG2 human hepatocyte cell line was obtained from the European Collection of Authenticated Cell Cultures and purchased from Sigma-Aldrich (Saint-Quentin-Fallavier, France − 85011430). During the maintenance phase, HepG2 cells were cultured in Dulbecco’s modified Eagle’s medium (Invitrogen, Carlsbad, CA, USA) supplemented with 10% fetal bovine serum (Invitrogen) and 1% penicillin/streptomycin (Life Technologies, Villebon-Sur-Yvette, France). All cell cultures were maintained at 37 °C in an atmosphere of 5% CO2/95% air. To analyze the effects of TOTUM-448 metabolites, the cells were preincubated for 24 h in DMEM with 10% human serum (either naïve, NHS or containing circulating metabolites, EHS) according to Clinic’n’Cell methodology (DIRV INRAE 18–0058), followed by an additional 48-hour incubation in a palmitate-induced lipidic stress environment (palmitate 250 µM; Sigma, Saint-Quentin-Fallavier, France).

### Palmitate solution

Palmitate (Sigma, Saint-Quentin-Fallavier, France) was first coupled to bovine serum albumin (BSA; Sigma, Saint-Quentin-Fallavier, France) and then fully dissolved in pure ethanol at 70 °C to achieve a final concentration of 500 mmol/L. This stock solution was mixed with a prewarmed BSA solution (10% w/w, 37 °C) to reach a final concentration of 5 mmol/L. The mixture was clarified by incubating at 55 °C for 15 min twice. The final palmitate: BSA molar ratio was set at 3.2:1. The control vehicle was prepared under the same conditions. A 10% w/w BSA solution was added to an equivalent volume of ethanol to match the final palmitate solution. The final ethanol concentration was less than 0.05% by volume in all experiments.

### Cell viability

Cell viability was assessed using an XTT-based method (Cell Proliferation Kit II, Sigma-Aldrich, Saint-Quentin-Fallavier, France). Experimental procedures were followed according to the supplier’s recommendations. Optical density was measured at 450 nm, with measurements performed in hexaplicate for each sample condition of the ten volunteers.

### DCF-DA staining

HepG2 cells were seeded on a 96-well dark-wall clear-bottom plate at a density of 12,000 cells/cm². Twenty-four hours after palmitate (PA) stimulation, cells were washed and incubated with 5 µM of 2′,7′-dichlorofluorescin diacetate (DCF-DA) solution (ab113851, Abcam) for 45 min at 37 °C in the dark, then rinsed with the dilution buffer according to the manufacturer’s protocol. Fluorescence was measured using a fluorescence plate reader (Berthold − Mitras) at Ex/Em = 485/535 nm in end-point mode.

### Red oil staining

Oil Red O solution (0.5% in isopropanol) was obtained from Sigma (Saint-Quentin-Fallavier, France) and staining was performed according to the supplier’s recommendations. The working solution (0.2% in 60% isopropanol) was prepared by mixing Oil Red O solution with distilled water in a 3:2 ratio. Prior to staining, cells were washed twice with PBS and fixed with 4% paraformaldehyde for 30 min at room temperature. After discarding the PFA solution, cells were washed twice with water, incubated with 60% isopropanol for 5 min, and then stained with the working Oil Red O solution for 20 min. After five additional washes with water, cells were observed under a microscope.

### Mono-, di- and triglycerides levels

Mono-, di- and triglyceride content in HepG2 cells was determined using a triglyceride assay kit (Abcam, Paris, France—ab65336) according to the manufacturer’s protocol. Tri-, di- and monoglycerides were converted to free fatty acids and glycerol, and the glycerol was oxidized to generate a colorimetric product measured at 570 nm. The lipase used in the kit has a high affinity for triglycerides in micellar form but a weaker affinity for di- and monoglycerides. However, since the enzyme cleaves triglycerides sequentially into free fatty acids and glycerol, all three forms may be detected.

### Cholesterol levels in HepG2 cells

Cholesterol levels were evaluated in cell lysates using a cholesterol quantification kit (Sigma, Saint-Quentin-Fallavier, France—MAK043) according to the manufacturer’s recommendations. Total cholesterol concentration was measured by a coupled enzyme reaction resulting in a fluorometric (λex = 535 nm/λem = 587 nm) product related to the cholesterol content.

### Real-Time RT-qPCR

mRNA from HepG2 cells were isolated using TRIzol™ Reagent (Ambion – Life Technologies) according to the supplier’s recommendations. The expression levels of DGAT2 (Diacylglycerol O-acyltransferase 2), SREBP1-c (Sterol regulatory element-binding protein 1c), CXCL-1 (CXC motif chemokine ligand 1), IL-1β, IL-6, MCP-1, TNFα, CHOP (C/EBP homologous protein), and XBP-1 mRNA were measured by RT-qPCR (PowerUp SYBRgreen, Applied Biosystems). β-Actin was used as a housekeeping gene. Primers were designed as follows: IL6-F: 5’- TTC TGT GCC TGC AGC TTC − 3’; IL6-R: 5’- GCA GAT GAG TAC AAA AGT CCT GA −3’; MCP-1-F: 5’- GCC TCT GCA CTG AGA TCT TC −3’; MCP-1-R: 5’- AGC AGC CAC CTT CAT TCC − 3’; TNFa-F: 5’- TCA GCT TGA GGG TTT GCT AC −3’; TNFa-R: 5’- TGC ACT TTG GAG TGA TCG G −3’; IL1b-F: 5’- GAA CAA GTC ATC CTC ATT GCC − 3’; IL1b-R: 5’- CAG CCA ATC TTC ATT GCT CAA G −3’; CXCL1-F: 5’- TCT CTC TTT CCT CTT CTG TTC CTA − 3’; CXCL1-R: 5’- CAT CCC CCA TAG TTA AGA AAA TCA TC −3’; DGAT2-F: 5’- TCA GCA GGT TGT GTG TCT TC −3’; DGAT2-R: 5’- GGC TGG TGT TTG ACT GGA A −3’; SREBP1c-F: 5’- CGG AAC CAT CTT GGC AAC AGT − 3’; SREBP1c-R: 5’- CGC TTC TCA ATG GCG TTG T −3’; XBP1-F: 5’- CGC TGT CTT AAC TCC TGG TTC − 3’; XBP1-R: 5’- CTG GAA CAG CAA GTG GTA GA −3’; CHOP-F: 5’- CAA TGA CTC AGC TGC CAT CT −3’; CHOP-R: 5’- AGC GAC AGA GCC AAA ATC AG −3’; ACTβ-F: 5’-ATT GGC AAT GAG CGG TTC-3’; ACTβ-R: 5’-GGA TGC CAC AGG ACT CCA-3’.

### Cell Lysis

The lysis buffer was prepared by mixing 50 mmol/L Tris pH 7.8, 150 mmol/L NaCl, 0.5% sodium deoxycholate, and 1% NP40. Cell lysates were stored at − 80 °C until analysis.

### Protein quantification

Protein content was measured using the BCA Protein Assay Kit (Sigma-Aldrich, Saint-Quentin-Fallavier, France). The BCA protein assay is based on a biuret reaction, where the reduction of Cu2 + to Cu + in the presence of proteins in an alkaline environment is proportional to the protein concentration. The chromogenic reagent bicinchoninic acid chelates the reduced copper, forming a purple complex that absorbs at 562 nm.

### Caspase-3 activity

Caspase-3 activity in cells was evaluated using the Caspase-3 Assay Kit (Abcam, Paris, France—ab39401) according to the manufacturer’s protocol. To lyse the frozen monolayer of HepG2 cells grown on a twelve-well plate, 200 µL of the provided lysis buffer was used per well. Fifty microliters of freshly prepared lysate were then mixed with 55 µL of reconstituted reaction mix. Optical density at 405 nm was measured every two minutes for 90 min at 37 °C.

### ATF6 activity

Nuclear extracts were prepared from HepG2 cells using the Nuclear Extraction Kit (Abcam, Paris, France—ab113474), and protein content was determined using the BCA Protein Assay Kit (Sigma-Aldrich, Saint-Quentin-Fallavier, France). ATF6 protein expression was assessed using the ATF6 ELISA Kit (Signosis, TE-0041) according to the manufacturer’s instructions.

### Statistics

Statistical analyses and figure generation were performed using Prism V.10.4.1 (GraphPad Software). The statistical plan included a Shapiro–Wilk normality test to determine if the data were consistent with Gaussian distribution. If the data were not normally distributed, a Kruskal–Wallis non-parametric test was used, followed by Dunn’s test for post hoc comparisons. For normally distributed data with equal variance, one-way or two-way ANOVA with Tukey’s test for multiple comparisons was applied. For inflammatory gene expression, a two-way ANOVA targeting a main column effect was followed by Tukey’s post hoc test. If data were missing, making it impossible to run a repeated-measure two-way ANOVA, a mixed-effects analysis was used instead. In Fig. 1, values are presented as means ± SEM, with statistical significance indicated as follows: * for *p* < 0.05; ** for *p* < 0.01; *** for *p* < 0.001; **** for *p* < 0.0001; ns for *p* > 0.05. For Figs. 2, 3, 4 and 5, values are presented as box plots indicating median and interquartile range (lower and upper), with whiskers indicating minimum and maximum values. Statistical significance is indicated as follows: * for *p* < 0.05; ** for *p* < 0.01; **** for *p* < 0.0001; ns for *p* > 0.05.

## Supplementary Information

Below is the link to the electronic supplementary material.

Supplementary Material 1

## Data Availability

The datasets generated and analyzed during the current study are available from the corresponding author on reasonable request.
