# Supplementary material for: Bioavailable human metabolites from TOTUM-448 (plant-based formulation) maintain liver cell functionality in a hyperlipidic context that drives MASLD onset
Source: Sci Rep. 2025 Dec 16;16:2748. doi: 10.1038/s41598-025-32556-z (PMC12824265; doi:10.1038/s41598-025-32556-z)
Supplement: Supplementary file 1 — Supplementary Material 1 [file 41598_2025_32556_MOESM1_ESM.docx]

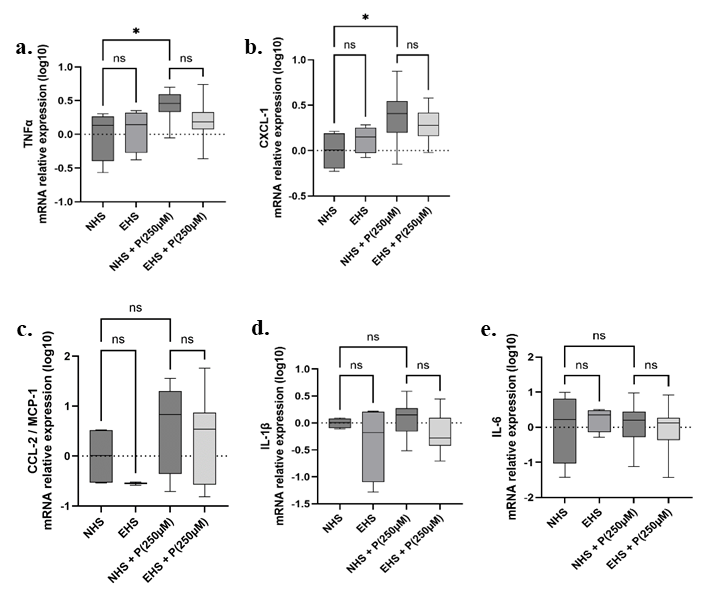


**Supplemental Figure S1.** Inflammatory gene expressions analyzed separately.

mRNA relative expression of CXCL1 IL-1β, IL-6, MCP-1 and TNFα (A, B, C, D, E and F, respectively). Measurements were realized in triplicate for each volunteer (n=10 volunteers). Boxes indicate median and interquartile range (lower and upper), while whiskers indicate minimum and maximum *: p < 0.05; ns: p > 0.05.

| **Compound types (sorted by families)** | **Extract content (g/100 g)** |
| --- | --- |
| Total sugars | 27.1 |
| Total lipids | 12.6 |
| Total Proteins | 0.8 |
| Insoluble dietary fiber | 2.36 |
| Choline* | 13.67 |
| Total phenolic compounds | 8.7 |
| Total anthocyanins | 0.536 |
| Monocaffeoylquinic acids |  |
| Chlorogenic acid | 0.517 |
| Cryptochlorogenic acid | 0.324 |
| Neochlorogenic acid | 0.319 |
| Other monocaffeoylquinic acids | 0.115 |
| Dicaffeoylquinic acids |  |
| Cynarine | 0.229 |
| 4,5-Dicaffeoylquinic acid | 0.098 |
| 3,5-Dicaffeoylquinic acid | 0.074 |
| 3,4-Dicaffeoylquinic acid | 0.056 |
| Caffeic acid | 0.008 |
| Oleuropein | 6.223 |
| Oleuropein isomers | 0.757 |
| Ligstroside | 0.131 |
| Luteolin | 0.017 |
| Luteolin-7-O-glucoside | 0.880 |
| Luteolin-7-O-glucuronide | 0.277 |
| Luteolin-4-O-glucoside | 0.083 |
| Apigenin-7-O-glucoside | 0.062 |
| Apigenin-7-O-glucuronide | 0.139 |
| Apigenin-7-O-rutinoside | 0.037 |
| Verbascoside | 0.152 |
| Terpenes and terpenoids |  |
| Oleanolic acid | 0.199 |
| Cynaropicrin | 0.139 |
| Saponins |  |
| Chrysanthellin A | 0.133 |
| Chrysanthellin B | 0.215 |
| Alkaloids |  |
| Piperin | 0.044 |

* in choline chloride equivalent.

**Supplemental Table S1: chemical characterization of TOTUM-448**

Table S1 shows the chemical characterization of TOTUM-448. Total phenolic compound levels (in gallic acid equivalent), total sugars level (in glucose equivalent), and total fat levels (in sunflower oil equivalent) were respectively assessed using the Folin-Ciocalteu colorimetric method, the Dubois colorimetric method, and the sulfo-phospho-vanillin (SPV) colorimetric assay. A fluorometric method using fluoraldehyde *o*-phthaldialdehyde reagent (OPA) was used to quantify the protein content, and the Lee method was used to quantify insoluble dietary fibers. A more precise characterization of phytochemical compounds was performed by HPLC-UV/Visible/RID-MS using 1260 LC system and 1200 LC system with a 6110 Single Quad MS-ESI detector (Agilent Technologies, Santa Clara, CA, USA) with a C18 Prodigy reversed-phase column (250 mm × 4.6 mm, 5 μm; Phenomenex, USA) and an Atlantis HILIC Silica column (150×4.6 mm, 5 μm, Waters, The Netherlands).

| **Compound Name** | **Precursor Ion (m/z)** | **Product Ion (m/z)** | **Fragmentor** | **Collision Energy (V)** | **Cell Accelerator Voltage (V)** | **Polarity** |
| --- | --- | --- | --- | --- | --- | --- |
| Hydroxytyrosol sulfate | 233 | 153 | 120 | 10 | 5 | Negative |
| Homovanillic acid sulfate | 261 | 181 | 100 | 10 | 5 | Negative |
| Ferulic acid sulfate | 273 | 193 | 100 | 10 | 5 | Negative |
| Tyrosol glucuronide | 313 | 137 | 120 | 17 | 5 | Negative |
| Hydroxytyrosol glucuronide | 329 | 153 | 120 | 12 | 5 | Negative |
| Oleuropein glucuronide | 553 | 377 | 100 | 10 | 5 | Negative |
| Luteolin glucuronide | 461 | 285 | 85 | 10 | 5 | Negative |

**Supplemental Table S2: MRM transitions for phenolic acid analysis in serum**
